# Histochemical Analysis of Paraspinal Rotator Muscles From Patients With Adolescent Idiopathic Scoliosis: A Cross-Sectional Study

**DOI:** 10.1097/MD.0000000000000598

**Published:** 2015-02-27

**Authors:** Marcelo Wajchenberg, Delio Eulalio Martins, Rafael de Paiva Luciano, Eduardo Barros Puertas, David Del Curto, Beny Schmidt, Acary Bulle de Souza Oliveira, Flavio Faloppa

**Affiliations:** From the Universidade Federal de Sao Paulo (UNIFESP/EPM), Rua Borges Lagoa, Vila Clementino, Sao Paulo, Brazil.

## Abstract

Morphological, biochemical, and histopathological alterations in the paraspinal skeletal muscle of patients with adolescent idiopathic scoliosis (AIS) have been extensively reported. We evaluated rotator muscle fibers from the apex vertebra of AIS patients through histological and immunohistochemical analysis.

A population of 21 female AIS patients who underwent corrective surgery between 2010 and 2013 had biopsies taken from the paraspinal muscle in the convex and concave sides of the thoracic curve apical vertebra. Serial sections were stained following routine protocols for hematoxylin and eosin (HE), Sudan red, Gomori trichrome, NADH, ATPase, and cytochrome oxidase. We assessed muscular atrophy and hypertrophy, fatty proliferation, endomysial and perimysial fibrosis, the presence of hyaline fibers, mitochondrial proliferation, muscular necrosis, nuclear centralization, and inflammation. Two independent professionals evaluated the slices.

The thoracic curves had an average Cobb angle of 68 degree. Comparative analysis of the concave and convex sides was performed with McNemar test at a significance level of 5%. Results showed significant differences in both endomysial and perimysial fibrosis and fatty involution between the two sides of the apex vertebra.

Paraspinal muscles in the concave side of the scoliosis apex had significantly more fibrosis and fatty involution. However, both sides showed signs of myopathy, muscular atrophy due to necrosis, presence of hyaline fibers, and mitochondrial proliferation.

## INTRODUCTION

The set of conditions named idiopathic scoliosis (IS) encompasses any lateral curvature of the vertebral column associated to the rotation of vertebral bodies, and with no known cause.^1^ Depending on the age of onset, IS might be infantile (0–3 years of age), juvenile (4–9), or adolescent (10 to adulthood).^2^

The etiology and pathogenesis of IS have long been objects of study. As early as 1882, Adams autopsied several cadavers with scoliosis, describing their deformities. He suggested that vertebral bone distortions preceded secondary muscular alterations, opposing contemporary authors who saw scoliosis primarily as a paraspinal muscle disorder.^3^ Since then, several factors have been proposed as potential causes for IS including abnormal growth patterns, structural tissue deficiency found in specific conditions and syndromes, asymmetrical growth of trunk and limbs, alterations in the sagittal vertebral column, and environmental factors such as food quality. Previous studies have also indicated that the disease might develop from a combination of genetic traits and environmental factors.^2,4^

Later studies showed an association between morphological, histopathological, and biochemical alterations in paraspinal muscles specifically in adolescent IS (AIS). The most frequently reported abnormalities referred to increased type I fibers in the convex side and loss of type II fibers in the concave side of the curvature,^5,6^ elevated concentrations of intracellular glycogen and lipids,^7^ structural changes in the sarcolemma and the myotendinous junction^8,9^; alterations in muscle enzymatic activity^10^; and increased intracellular calcium concentrations.^11^

Despite this extensive literature, the etiology of AIS remains obscure. Studies focused on muscular alterations often meet with difficulties in sample collection, and others usually have a bias toward the neuropathic aspects of the disease. To shed light on the subject and evaluate the hypothesis that the disease is primarily myopathic, we have analyzed in detail profound biopsies of the rotator muscles in the apex of the deformity through histological and immunohistochemical analyses.

## MATERIALS AND METHODS

The present study was conducted through biopsy analysis from 21 female AIS subjects with normal body mass index and no associated comorbidities. Patients were all surgically treated and followed at the same outpatient facility from May 2010 to June 2013 with a mean age of 14.8 years (Table 1). The work was approved by the Committee on Research Ethics (no. 639.087) of our Institution and all patients or legal guardians, in the case of minors, voluntarily signed an informed consent form.

**TABLE 1:**
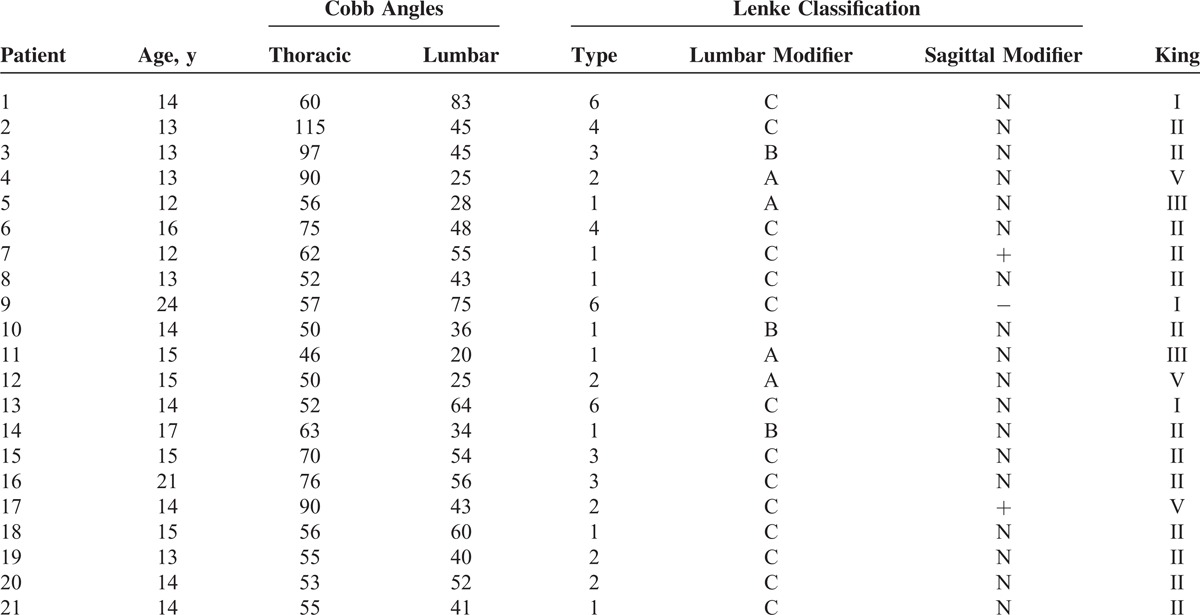
Characteristics of the Patients’ Scoliotic Curves

During corrective surgeries, biopsies from rotator muscles in both sides of the scoliosis apex vertebra were obtained from all patients. Muscle biopsy followed the procedure described by Schmidt et al,^12^ and extracted samples were protected in gauze and refrigerated. Samples were immediately taken to the laboratory, where they were placed over a cork, embedded in gum tragacanth, and covered in talc. Samples were then immersed in liquid nitrogen for 20 s, and the blocks were stored at −80°C. After serial cryostat sectioning of the blocks, standard staining techniques included hematoxylin and eosin (HE), Sudan red, Gomori trichrome, cytochrome oxidase, ATPase and NADH.

Samples were analyzed for muscular atrophy and hypertrophy, fatty proliferation, endomysial and perimysial fibrosis, presence of hyaline fibers, mitochondrial proliferation, muscular necrosis, nuclear centralization, type grouping, presence of central core myopathy, and inflammation (Table 2). Two independent professionals evaluated each parameter, and there were no cases of disagreement between the two regarding the analyses. Necrosis, atrophy, hypertrophy, fatty involution, and endomysial, perimysial, and hyaline fibroses were classified as: absent; scarce (<25%); mild (<50%); moderate (<75%); and severe (>75%). Incidence of a determined condition was considered as the sum of moderate and severe cases for each sample.

**TABLE 2:**
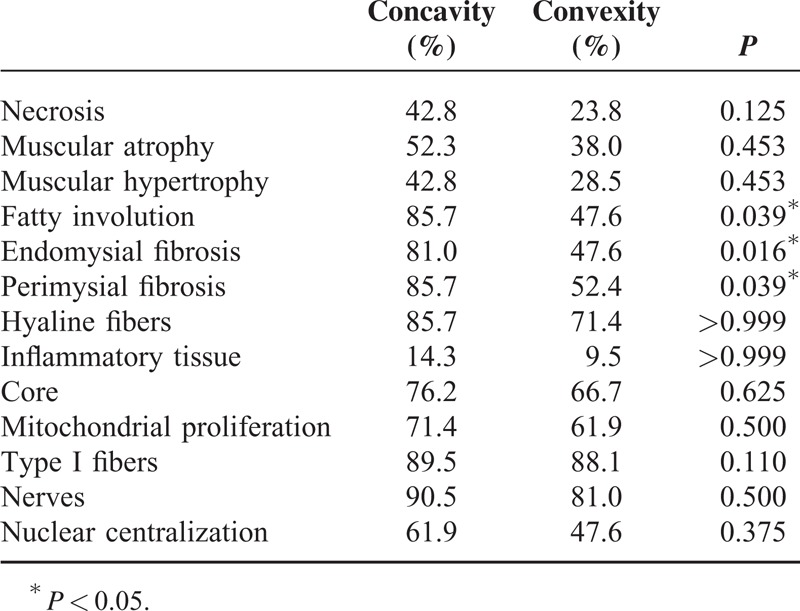
Histopathological Alterations in the Paraspinal Skeletal Muscle of Patients With Adolescent Idiopathic Scoliosis

## RESULTS

Table 1 summarizes data from patients included in the study, and includes age distribution, average Cobb angles, and Lenke and King classification. Only four cases (19%) presented with the main curvature in the lumbar region; among them, patient 9 was the only one to have hypokyphosis (Cobb angle <10°). Hyperkyphosis (Cobb angle >40°) was detected in two patients (9.5%). The average Cobb angle was 68° for the main thoracic curve and 77.5° for the thoracolumbar and lumbar curves.

**FIGURE 1:**
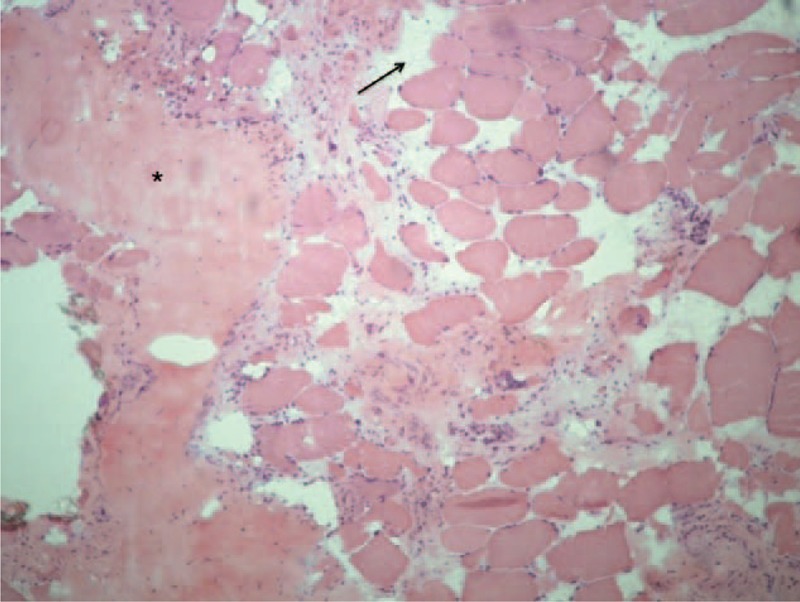
Hematoxylin and eosin stain of rotator muscle tissue from an adolescent idiopathic scoliosis patient showing areas of endomysial and perimysial fibrosis as well as fatty proliferation.

**FIGURE 2:**
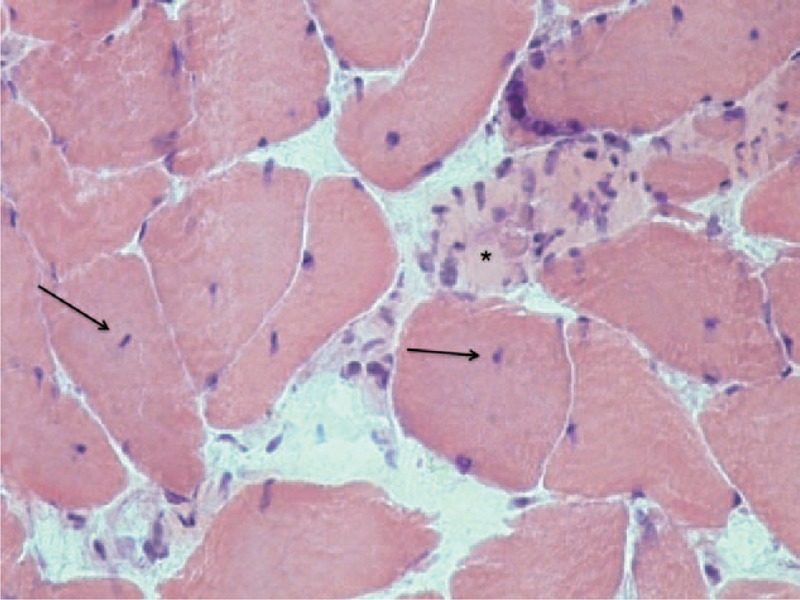
Hematoxylin and eosin stain of rotator muscle tissue from an adolescent idiopathic scoliosis patient showing areas of muscular necrosis and nuclear centralization.

**FIGURE 3:**
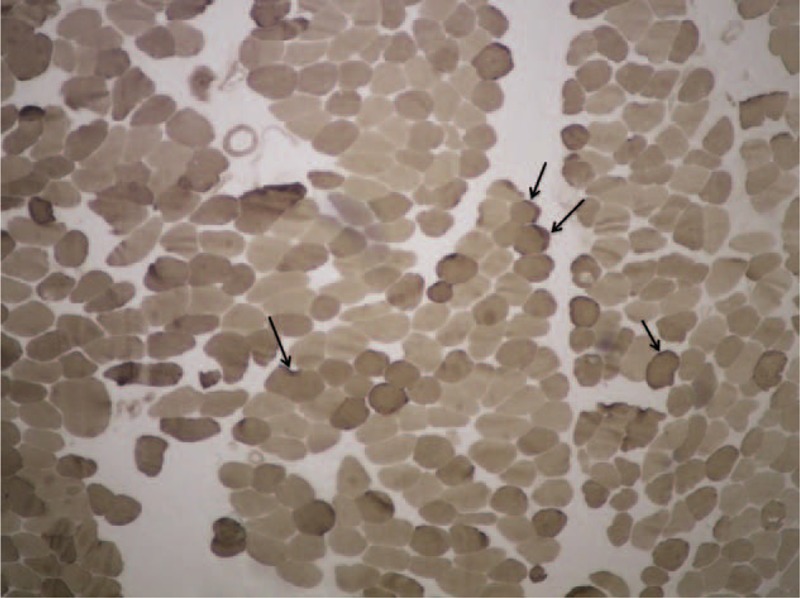
Cytochrome oxidase stain of rotator muscle tissue from an adolescent idiopathic scoliosis patient showing muscular atrophy and mitochondrial proliferation.

**FIGURE 4:**
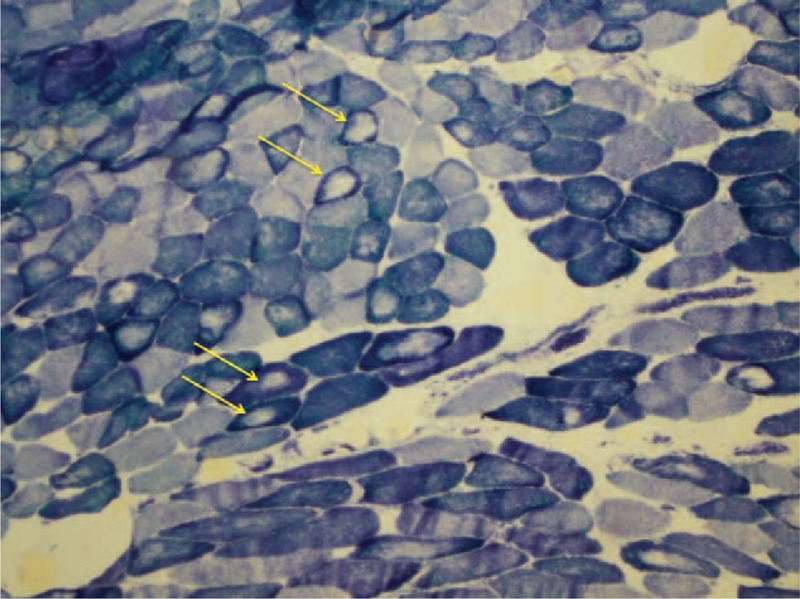
NADH stain of rotator muscle tissue from an adolescent idiopathic scoliosis patient showing central core lesion of the muscle fiber.

Comparative analysis of the concave and convex sides of each sample was performed with McNemar test at a significance level of 5%. Relative frequencies found and *P* values are presented in Table 2. Endomysial and perimysial fibrosis as well as fatty involution were significantly greater in the concave side of the apex vertebra (Table 2 and Figure 1). We were also able to identify other alterations, albeit with no significant differences between curvature sides. These included hyaline fibers, muscle necrosis, and nuclear centralization (Figure 2); inflammation, muscular atrophy, and mitochondrial proliferation (Figure 3); and low-oxidative areas in the muscle fibers suggestive of central core myopathy (Figure 4).

## DISCUSSION

The etiology and pathogenesis of IS have yet to be unveiled. Currently, there are several parallel and overlapping hypotheses involving genetic, structural, and environmental factors. An environmental role becomes evident in studies that show only a partial fit of curvatures between homozygote twins, which can vary with disease severity.^13^ A few previous studies propose an association between AIS and muscular disorders. Since 1882, Adams attempted to connect primary dorsal torsion, including gibbosities, with a secondary alteration in the paraspinal muscles of IS patients. IS becomes more evident after puberty during adolescence, when patients show greater axial growth. The progression of deformities characteristic of IS makes it essential that we further understand the disorders that rotator paraspinal muscles undergo during the disease. The best way to assess these disorders is through biopsies taken during surgery. Previous studies conducted with percutaneous biopsies or through electromyography could not adequately isolate the proper muscle fibers.

We analyzed muscle fibers, bilaterally, in the deformity apex of AIS patients who underwent biopsy during corrective surgery. Fibrosis and fatty involution were significantly greater in the concave side of the scoliosis. Both sides showed muscular atrophy, necrosis, hyaline fibers, mitochondrial proliferation, and areas suggesting central core lesion. In our samples, we could not find evidence of a primarily neurogenic disease as previously reported by Chagas et al,^14^ who found fascicle-type grouping in IS patient biopsies.^14^

Our findings, along with previous studies, suggest that AIS might be related to congenital myopathies. This type of hereditary myopathy is characterized by precocious muscular alterations starting in infancy, with stable or slowly progressing effects.^15^ Congenital myopathy morbidity is associated with the involvement of respiratory muscles, adding to the orthopedic problems such as scoliosis and contractures. In the last decade, there have been significant advances in the identification of genetic myopathies, although several poorly defined diseases still remain with no known association to a specific genetic mutation. Central core disease, first described in 1956 by Shy and Magee,^16^ is a congenital dominant autosomal myopathy with rare recessive cases described. The original study reported a family case in which the dominant trait was described as muscle fibers with an abnormal central zone, lacking in oxidative enzymes. These characteristics were significantly observed in our samples.

It is worth mentioning that patients in need of surgery, such as the ones in our study, might have a more severe deformity on average. In turn, this may result in more advanced myopathies and greater damage to the concave side, causing the characteristic IS torsion. These ideas can only be validated as more samples are analyzed and comparisons are made with biopsies from healthy individuals.

Furthermore, we cannot deduce from our data alone that the observed myopathies are primary and have a causal relationship with the disease. Other muscle groups might also come into play, as reported by Sahgal et al, who performed biopsies of the gluteus muscles.^17^ Further studies are needed, therefore, to probe into genetic factors and systemic muscular diseases that might trigger the observed abnormalities in the paraspinal rotator muscles.

All procedures performed in studies were in accordance with the ethical standards of the institutional and national research committee and with the 1964 Helsinki declaration and its later amendments or comparable ethical standards.

## References

[1] Kane WJ Newer knowledge of scoliosis: a tribute to John H. Moe. M.D. *Clin Orthop Relat Res* 1977; 126:2–3. 340091

[2] Wynne-Davies R Familial (idiopathic) scoliosis. A family survey. *J Bone Joint Surg Br* 1968; 50:24–30. 5641594

[3] Adams W Lectures on the pathology and treatment of lateral and other forms of curvature of the spine. 2nd ed. London: J. & A. Churchill; 1882.

[4] Riseborough EJ Wynne-Davies R A genetic survey of idiopathic scoliosis in Boston, Massachusetts. *J Bone Joint Surg Am* 1973; 55:974–982. 4760104

[5] Fidler M Jowett R Troup J Zorab P Histochemical study of the function of multifidus in scoliosis. *Scoliosis and Muscle*. London: William Heinemann Medical Books; 1974 184–192.

[6] Maffulli N Histochemical and physiological studies in idiopathic scoliosis. *Ital J Orthop Traumatol* 1990; 16:61–71. 2116383

[7] Wong Y Yau A Low W Ultrastructural changes in the back muscles of idiopathic scoliosis. *Spine (Phila Pa 1976)* 1977; 2:251–260.

[8] Khosla S Tredwell SJ Day B An ultrastructural study of multifidus muscle in progressive idiopathic scoliosis. Changes resulting from a sarcolemmal defect at the myotendinous junction. *J Neurol Sci* 1980; 46:13–31. 7373342 10.1016/0022-510x(80)90040-4

[9] Ovalle WK Tredwell SJ The paraspinal myotendinous junction: a possible morphological marker for idiopathic scoliosis. *Orth Trans* 1983; 7:4.

[10] Cotic V Bizjak F Turk V The activity of proteinases of the paravertebral muscles in idiopathic scoliosis. Scoliosis and Kyphosis. M Pecina, Dubrovnik; 1983: p. 250.

[11] Blatt J Rubin E Botin G Impaired calcium pump activity in idiopathic scoliosis. Possible etiological role of a membrane defect. *Orth Trans* 1984; 8:143.

[12] Schmidt B Gabbai A Oliveira A Biópsia muscular, nova metodologia: a dança dos “farabeufs. *Rev Bras Ortop* 1988; 23:21–26.

[13] Wajchenberg M Martins DE Puertas EB Aspectos genéticos da Escoliose Idiopática do Adolescente. *Coluna/Columna* 2012; 11:234–236.

[14] Chagas JCM Schimidt B Puerta EB Estudo histoquímico dos músculos rotadores do dorso em pacientes com escoliose idiopática do adolescente. *Rev bras ortop* 1998; 33:111–118.

[15] Dubowitz V Neuromuscular disorders in childhood. Old dogmas, new concepts. *Arch Dis Child* 1975; 50:335–346. 1103747 10.1136/adc.50.5.335 PMC1544403

[16] Dubowitz V Platts M Central core disease of muscle with focal wasting. *J Neurol Neurosurg Psychiatry* 1965; 28:432–437. 5838477 10.1136/jnnp.28.5.432 PMC495930

[17] Sahgal V Shah A Flanagan N Morphologic and morphometric studies of muscle in idiopathic scoliosis. *Acta Orthop Scand* 1983; 54:242–251. 6846001 10.3109/17453678308996564

